# Supplementary material for: Retention rate of vaccination card and its associated factors among vaccinated children aged 12─23 months in Ethiopia: Multilevel logistic regression analysis
Source: PLoS One. 2024 Jul 11;19(7):e0306421. doi: 10.1371/journal.pone.0306421 (PMC11238995; doi:10.1371/journal.pone.0306421)
Supplement: Attachment — Submitted filename: Response to Reviewers.docx [file pone.0306421.s001.docx]

**Authors’ response to reviews**

# Title: Retention rate of vaccination cards and associated factors among vaccinated children aged 12-23 months in Ethiopia, multilevel logistic regression analysis

Authors:

Abiyu Abadi Tareke ([abiyu20010@gmail.com](mailto:abiyu20010@gmail.com))

Atikaw Tewabe Ayelign

Thomas Kidanemariam Yewodiaw

Enyew Woretaw Shiferaw

Habitu Birhan Eshetu

Ermias Bekele Enyew

Version: 1

Date: March 31, 2024

Point by point response for editors/reviewers’ comments

Manuscript number: PONE-D-23-21113

Dear editor/reviewer:

Dear all,

We express our profound appreciation for the insightful and productive feedback that you have provided. Your invaluable comments have significantly enriched the quality of the manuscript, and have greatly augmented our expertise in the realm of scientific paper writing. The authors have diligently considered each of the comments and queries raised by the editors and reviewers, and have responded to them in a targeted manner. Our comprehensive point-by-point rejoinders to all the comments and questions can be found in the subsequent pages. In addition, an accompanying supplementary document has been enclosed, which showcases the modifications made in detail, using the track changes feature. We also made some change to fix grammatical error in some paragraphs.

**Review Comments to the Author**

**Reviewer #1:** 
**Reviewer’s comment:** 1. Title:
i. Line number 1-3: Avoid the full stop from the title.

**Authors’ response:** Thank you for your feedback. We appreciate your suggestion to avoid using a full stop in the title. We made the necessary revisions accordingly.
2. Abstract:
**Reviewer’s comment:** i. Start a sentence with a capital letter! for example, the first sentence of the background (line number 17) and conclusion (line number 31) began with small letter.

**Authors’ response:** Thank you for pointing out this oversight. We apologize for the inconsistency and we ensured that all sentences begin with capital letters as appropriate, including the first sentences of the background and conclusion sections.
**Reviewer’s comment:** ii. The background does not show the knowledge gap.

**Authors’ response:** Thank you for your feedback. We acknowledge the importance of clearly demonstrating the knowledge gap in the background section. We revised the background to explicitly highlight the gap in understanding regarding the retention rate of vaccination cards and its associated factors in Ethiopia.
**Reviewer’s comment:** iii. Line number 21: At the end of the objective, what the term "..., in 2016" refers?

**Authors’ response:** Thank you for bringing this to our attention. The phrase "in 2016" at the end of the objective does not seem to have a clear connection or relevance. We reviewed and revised the objective statement to ensure clarity and coherence as “*This research aimed to assess the retention rate and associated factors of vaccination cards in Ethiopia, using data of 2016 Ethiopian demographic health survey.* ”
**Reviewer’s comment:** iv. Line number 23: report your outcome variable with its 95% CI!

**Authors’ response:** Thank you for your comment. We appreciate your suggestion to report the outcome variable with its 95% confidence interval (CI). We ensured that the outcome variable is presented along with its corresponding confidence interval in the results section of our manuscript. See the revised version of the manuscript.

**Reviewer’s comment:** v. Line number 23: the term "...fraction of their guardians..." is not appropriate. Additionally, it is not appropriate to use a term "approximately" while you reported the exact/actual figure (684)

**Authors’ response:** Thank you for your feedback. We acknowledge that the term "fraction of their guardians" is not be the appropriate phrase to describe human being and can lead to confusion. We revised the language to provide a clearer description of the population. Additionally, we recognize that using "approximately" when reporting an exact figure is unnecessary. We removed the term "approximately" and report the exact figure of 684 accordingly and we re-written it as “Among the cohort of 1,304 (weighted) immunized children, it was observed that 684 (52.5%, 95% CI: 49.7% to 55.2%), were able to bring their respective vaccination records”

**Reviewer’s comment:** vi. Line number 31: The conclusion for the outcome variable is not a conclusion rather it is a result.

**Authors’ response:** Thank you for your comment. We acknowledge your observation regarding the conclusion for the outcome variable. upon revising the sentence, we understand that the sentence is summary rather than conclusion. We revised the conclusion section to ensure that it provides a proper conclusion based on the results obtained from the study.

3. Introduction:t
**Reviewer’s comment:** i. Line number 23: Avoid a 'full stop' from the title.

**Authors’ response:** thanks for your valuable comment. We corrected accordingly.
**Reviewer’s comment:** ii Line number 42: Use the full version of "VC" instead of the acronym when you use for the first time!

**Authors’ response:** Thank you for your suggestion. We ensured to use the full term "vaccination card" instead of the acronym "VC" when mentioning it for the first time in the manuscript.
**Reviewer’s comment:** iii. Line number 64 & 65: authors documented that 'the rate of documentation practices related to routine immunization health cards of children is not measured'. If this is the knowledge gap, this would have been addressed in your study instead of card retention rate.

**Authors’ response:** Thank you for highlighting this point. We acknowledge that the rate of documentation practices related to routine immunization health cards like routine immunization register, appointment card, tally sheet and other immunization related documentation at facilities level is indeed an important knowledge gap. Our intention in including this facility-level problem in this paragraph is to illustrate the potential severity of the issue for caregivers when it occurs even within well-organized government facilities. We recommend future researchers to consider addressing this aspect in future research to provide a more comprehensive understanding of immunization documentation practices among service providers. In the revised version of this manuscript, we removed the broad terminology “documentation practices” and replaced by rate of vaccination card retention rate and the ambitious sentences re-written as *“The retention rate of routine immunization vaccination cards is not measured and the number of published articles concerning the VC retention rate of caregivers and associated factors in Ethiopia are limited.”*
**Reviewer’s comment:** iv. Line number 65 & 66: The sentence needs language edition.
Generally, what is the programmatic importance of keeping the vaccination cards after completion of the immunization? Are clients routinely informed to keep it after completion of vaccination??

**Authors’ response:** Thank you for your comment. We recognize the need for language editing in the sentence. The programmatic importance of keeping vaccination cards after completion of immunization lies in their role as crucial documentation for tracking vaccination history, ensuring timely and appropriate follow-up doses, and providing evidence of immunization status. Additionally, in Ethiopia, caregivers are typically instructed to retain their child's vaccination cards throughout the vaccination process and for the duration of their child's life after completing the vaccination schedule. However, the consistency of advising women to hold their child's vaccination cards may vary among health professionals and further investigation is required to determine whether clients are routinely informed to keep vaccination cards after completing their vaccination schedule in consistent way.

**4. Methods and materials:**
**Reviewer’s comment:** i. Line number 84: All under-five children could not be a source population as you couldn't infer your finding to all under five children. Use children age less than 2 years!

**Authors’ response:** Thank you for your suggestion. We made the necessary adjustment to specify the source population as "*children aged less than 2 years*" to accurately reflect the scope of our study findings.
**Reviewer’s comment:** ii. Line number 86: Since you noted, and we all know that that keeping vaccination card primarily benefits health care providers. What is your justification to excluded children age less than 12 months as long as they initiated the vaccination.

**Authors’ response:** Thank you for raising this point. We would like to clarify that children under 12 months of age were not intentionally excluded by us, but rather by the parameters of the survey. We acknowledge the significance of understanding vaccination practices and documentation among younger children and recognize the importance of exploring this further in future research endeavors.

**Reviewer’s comment:** iii. Line number 93-96: Since all the independent variables listed are individual level, why you planed and did a multilevel analysis?

**Authors’ response:** Thank you for your question. While it is true that the independent variables listed are at the individual level and we failed to list the community level factors in the first draft of the manuscript. we conducted a multilevel analysis to account for potential clustering effects within the data. In our study setting, there may be inherent variations in vaccination card retention rates across different communities that could influence the outcomes of interest. Therefore, by using a multilevel analysis approach, we aimed to appropriately model and address any potential clustering or contextual effects at the facility or community level, ensuring a more accurate assessment of the relationship between individual-level variables and vaccination card retention rates. In the revised version of the manuscript, we included the community level factors under the independent variable lists.
**Reviewer’s comment:** iv. Line number 103-108: Nothing is documented about the data collection tools.

**Authors’ response:** Thank you for bringing this to our attention. We apologize for the oversight. The data collection tools used in our study included structured questionnaires administered to caregivers to gather information on vaccination card retention and other relevant variables. We will ensure to provide a detailed description of the data collection tools in the Methods section of the manuscript.
**Reviewer’s comment:** v. Line number 106 or Line number 171: anyone can access the data if he/she get the link from someone else. So, did you attach an authorization letter/text from the responsible body for this particular study? You need to attach it as a supplementary file.

**Authors’ response:** As authors, we thank the reviewer for their valuable feedback. We understand the importance of ensuring proper authorization for accessing the data used in our study. Nevertheless, the DHS Demographic Health Survey grants access to the datasets without the need for repeated authorization requests for each study.

**Reviewer’s comment:** vi. Line number 108: What the number in bracket "(3)" refers?

**Authors’ response:** The number in parentheses "(3)" was a typographical error and does not have any significance or intended reference in the context of the manuscript. So we removed it.
**Reviewer’s comment:** vii. Line number 113: The description under the heading "Sample size and sampling procedures" is about sampling procedures, but nothing is stated about sample size estimation except mentioning a weighted sample size used which is a result, not the plan.

**Authors’ response:** We acknowledge the oversight regarding the description under the heading "Sample size and sampling procedures." We apologize for any confusion. We will revise the section to include information about the sample size estimation process, including the methods and rationale used for determining the weighted sample size. Thank you for bringing this to our attention.
**Reviewer’s comment:** viii. Line number 121: You used the 2016 EDH data but why you cited reference 14 and 15 which are the 2005 and 2011 EDHS?

**Authors’ response:** We apologize for the oversight in citing references 14 and 15, which pertain to the 2005 and 2011 EDHS data, respectively, while utilizing the 2016 EDHS data. This was an error in referencing, and we appreciate your attention to detail. We corrected this discrepancy and ensured that our citations accurately reflect the data used in our study. Thank you for bringing this to our attention.
**Reviewer’s comment:** ix. Line number 129: Did you get a community level factors that affect your outcome variable in your literatures review or clinical evidence which initiated you to consider multilevel analysis?

**Authors’ response:** While we did not specifically identify community-level factors directly associated with vaccination card retention in our literature review, the intracluster correlation coefficient (ICC) obtained from running the null model provided evidence supporting the rationale for conducting multilevel logistic regression. Additionally, previous studies have highlighted the importance of considering multilevel analysis when examining vaccination outcomes, particularly to account for the nested nature of individual-level data within communities. Therefore, based on both the literature review and clinical evidence, we deemed it appropriate to consider multilevel analysis to investigate the influence of community-level factors on our outcome variable.
**Reviewer’s comment:** x. Line number 160: Use the full version of "MOR" instead of the acronym when you use it for the first time!

**Authors’ response:** Thank you for your suggestion. We will ensure to use the full version of "MOR" (Median Odds Ratio) when it is mentioned for the first time in the manuscript
5. Results:
**Reviewer’s comment:** i. Line number 211: It would have been good if I can access table 3. It would have been good to mention all the independent variables that you considered under a community and individual level factors. This is because the community level factor specifically residence can be affected by individual level factors like educational status.

**Authors’ response:** Thank you for your comment. We apologize for any inconvenience caused by the inability to access Table 3. We will ensure that Table 3, along with all relevant independent variables considered under community and individual level factors, are clearly mentioned in the manuscript. We acknowledge the interplay between community and individual level factors, particularly regarding residence and educational status, and provided a comprehensive explanation of how these factors were accounted for in our analysis.
6. Discussion:
**Reviewer’s comment:** i. Line number 217: When you compare your finding with other studies (Nepal, India, Nepal, and Pakistan), their findings should be documented so that readers can see and approve the comparison decision. You have to cite the reference for the study done in Pakistan (line number 218).

**Authors’ response:** We appreciate the reviewer's feedback and acknowledge the importance of providing documentation for comparisons with other studies. In response to this comment, we have revised the manuscript to include proper citations for the studies conducted in Nepal, India, and Pakistan.
**Reviewer’s comment:** ii. Line number 220-222: Needs language revision including punctuation.

**Authors’ response:** Thank you for bringing this to our attention. We will thoroughly revise the language and punctuation in lines 220-222 to ensure clarity and correctness. We appreciate your feedback and will make the necessary improvements to enhance the readability of the manuscript.
**Reviewer’s comment:** iii. Line number 221-223: You explained that due to less educational level of the rural women, cards retention rate is low. If this so, did you consider educational level as independent variable and what was the result?

**Authors’ response:** Thank you for your query regarding the consideration of educational level as an independent variable in our analysis. We appreciate the opportunity to clarify our methodology. In our study, we indeed considered educational level as a potential independent variable and conducted statistical tests to assess its relationship with card retention rates. However, the results indicated that the relationship was non-significant.
**Reviewer’s comment:** iv. Line number 223-225: The explanation that rural women used the card for entertainment for their children is too poor.

**Authors’ response:** Thank you for your comment regarding the explanation provided for why rural women used the card for entertainment for their children. We acknowledge your concern and understand the importance of providing clear and comprehensive explanations in our research. Upon reflection, we agree that the explanation provided may not have sufficiently addressed the complexity of factors influencing the use of the card for entertainment purposes. In our revised manuscript, we endeavored to provide a more thorough and nuanced explanation, taking into account cultural, social, and economic factors that may contribute to this behavior among rural women.
**Reviewer’s comment:** v. Line number 226-232: The policy or programmatic implication of the finding that rural women have less retention rate is not clear and is not strong.

**Authors’ response:** Thank you for your comment regarding the explanation provided for why rural women used the card for entertainment for their children. We acknowledge your concern and understand the importance of providing clear and comprehensive explanations in our research. Upon reflection, we agree that the explanation provided may not have sufficiently addressed the complexity of factors influencing the use of the card for entertainment purposes. In our revised manuscript, we endeavored to provide a more thorough and nuanced explanation, taking into account cultural, social, and economic factors that may contribute to this behavior among rural women.
**Reviewer’s comment:** vi. Line number 223-225: duplication of idea.

**Authors’ response:** Thank you for your observation. We reviewed lines 223-225 to identify any duplication of ideas and streamline the content to ensure clarity and conciseness in conveying our message. We appreciated your feedback and will make the necessary adjustments accordingly.
**Reviewer’s comment:** vii. Line number 239: "... prevalence of VC possession rate." If you used the term "prevalence", do not need to use the term "rate"!

**Authors’ response:** Thank you for your comment. We appreciate your feedback. We acknowledge the redundancy in the term "prevalence of VC possession rate" and apologize for the oversight. We revised the manuscript accordingly to remove the unnecessary term "prevalence" from line 239 and other part of the manuscript.
**Reviewer’s comment:** viii. Line number 254-256: Language edition!

**Authors’ response:** Thank you for your comment. We apologize for any confusion. In lines 254-256, we ensured that the language edition is clearly specified. If there are any specific language editions that the reviewer suggests or prefers, we will take that into consideration and make the necessary adjustments.
Generally, the discussion is shallow!
7. Conclusion:
**Reviewer’s comment:** i. Line number 260: delete the number.

**Authors’ response:** Thank you for your feedback regarding the conclusion section. We acknowledge your suggestion to delete the specific percentage (52.5%) mentioned in line 260. We made the necessary revision accordingly to remove the numerical value while still conveying the main findings of the study. We appreciate your attention to detail and will ensure that the conclusion section accurately reflects the study's outcomes.
**Reviewer’s comment:** ii. Line number 261-264: The forwarded recommendation "...advisable to customize the interventional strategy..." should be specific. What interventional strategy is/are needed to mitigate the low retention rate?

**Authors’ response:** Thank you for your comment regarding the specificity of the recommended interventional strategy. We acknowledge the need for a more concrete approach. Based on our findings. See the revised version of our manuscript.

Reviewer #2: Summary:
The authors have selected an interested topic, however, they were unable to justify the findings. The article needs significant revisions. Major gaps were observed in methods, data analysis, and results. Authors are requested to revisit the article and made changes to make it technically sound and equivalent to other scientific literature. the below comments can help improve the quality of article.

**Authors’ response:** We sincerely appreciate the reviewer's thorough assessment of our article and their valuable feedback. We acknowledge the importance of addressing the major gaps identified in the methods, data analysis, results, and discussion sections to ensured the technical soundness and equivalence to other scientific literature.
Abstract:
**Reviewer’s comment:** 1. Methods section missing in the abstract. It is suggested that methods section should be added in abstract.

**Authors’ response:** We thank the reviewer for their constructive feedback. We acknowledge the importance of providing a concise summary of the methods in the abstract to enhance clarity and transparency. In response to the reviewer's suggestion, we revised the abstract to include a brief description of the methods employed in our study. This addition will ensure that readers have a clear understanding of the approach used to analyze vaccination card retention among children in Ethiopia. We appreciate the opportunity to improve the comprehensiveness of our abstract.
**Reviewer’s comment:** 2. Line 25-27: do authors mean to say that card retention is higher among urban population than rural? If so, rephrase the statement accordingly

**Authors’ response:** Thank you for your comment. Yes, the authors intended to convey that card retention is lower among the rural population compared to the urban population. In the time analysis we used urban category as reference and rural category a comparison group. Dear reviewer upon reviewing the sentence that you suggested as to rephrase, we understood that the meaning of our interpretation is the same. We also emphasize that the interpretation of their findings remains consistent with your understanding. Dear reviewer, if you have any further questions or suggestions, we are fully prepared to consider and address them accordingly.

**Reviewer’s comment:** 3. Line 35-36: better to add the desirable retention rate, if any

**Authors’ response:** Thank you for your suggestion regarding the addition of a desirable retention rate. We appreciate your input and recognize the importance of providing context for the retention rate discussed in our article. Upon consideration of your comment, we will revise the manuscript to include information about any desirable retention rate, if applicable.
Introduction:
**Reviewer’s comment:** 1. Line 41: its VPD not VSD

**Authors’ response:** We appreciate the reviewer's attention to detail and thank them for pointing out the error in our manuscript. We acknowledge that the correct abbreviation is VPD (Vaccine-Preventable Disease) rather than VSD. We made the necessary correction to ensure accuracy in our manuscript. Thank you for bringing this to our attention
**Reviewer’s comment:** 2. Line 42: VC? Please mention full form first. It is always good to avoid using self-generated abbreviations

**Authors’ response:** We appreciate the reviewer's suggestion regarding the use of abbreviations in our manuscript which is also raised by reviewer #1. We acknowledge that clarity is essential for readers, and we ensured to provide the full form of the abbreviation 'VC' (Vaccination Card) upon its first mention, followed by the abbreviated form for consistency throughout the manuscript. This adjustment will enhance the readability and comprehension of our study. Thank you for highlighting this concern.
**Reviewer’s comment:** 3. Line 45-57: not clear what information authors are trying to convey. Need to rephrase ensuring quality of information and coherence

**Authors’ response:** We appreciate the reviewer's feedback regarding the clarity and coherence of the section discussing the benefits of holding a vaccination card. We agree that this section could be revised to better convey the importance and usefulness of vaccination cards for different stakeholders. We rephrased this section to ensure that the information provided is of high quality and coherence, clearly highlighting how vaccination cards benefit parents, children, researchers, and care providers. Thank you for bringing this to our attention.
**Reviewer’s comment:** 4. Line 58-59: reference for the definition is missing. Also, please mention until when the card is expected to be saved by household? as “individuals who still have VC” seems unprecise

**Authors’ response:** We value the reviewer's feedback regarding the missing reference for the definition and the need for clarification regarding the duration for which vaccination cards are expected to be saved by households. We confirmed to provide the appropriate reference for the definition cited in the manuscript. Additionally, we acknowledge that the term 'individuals who still have VC' may be imprecise, and we revised the language to specify the expected duration for retaining vaccination cards by households. This adjustment will enhance the clarity and precision of our study. Thank you for bringing these concerns to our attention.
**Reviewer’s comment:** 5. Line 64-66: both statements are conflicting. There are articles available in the context of Ethiopia which have used vaccination cards to estimate the coverage. Authors should investigate the different search engines and explore

**Authors’ response:** Thank you for your valuable feedback. We appreciate your insight. In those lines, our intention was to convey the message that the study about vaccination card retention by families/caregivers is limited in Ethiopia, except for some studies that focus on documentation practices by healthcare providers in health facilities rather than in the community. Upon considering your comment, we have made some modifications. Please see the revised version of the manuscript for clarification.

**Reviewer’s comment:** 6. Significant revisions are required in the section. Authors are requested to revise the language and add relevant information based on available scientific information

**Authors’ response:** Thank you for your feedback regarding the introduction section of our manuscript. We appreciate the opportunity to improve the language and incorporate relevant information based on available scientific literature. We conducted a thorough revision of the introduction section to enhance clarity, coherence, and alignment with existing scientific knowledge. This involved refining the language to improve readability, providing additional background information to contextualize the study, and incorporating relevant references to support key points.
Methods:
**Reviewer’s comment:** 7. Sub-headings need revision, for instance, instead of study area mention study settings

**Authors’ response:** Thank you for your suggestion. We acknowledge the need for revision in the sub-headings. We updated "study area" to "study settings" to better reflect the content and improve clarity for readers.
**Reviewer’s comment:** 8. References missing in information provided under ‘study area’ sub-head

**Authors’ response:**
**Reviewer’s comment:** 9. Under ‘Study area’ it would be better if relevant statistics are provided such as information regarding fertility rate, current immunization rate etc.

**Authors’ response:** We appreciate the reviewer's suggestion. In response to this feedback, we have updated the 'Study Area' section to include relevant statistics such as the fertility rate and current immunization rate. This addition aims to provide a more comprehensive overview of the demographic and healthcare landscape within the study area.
**Reviewer’s comment:** 10. Line 97: only one definition is provided

**Authors’ response:** Dear reviewer thank you for your comment. We appreciate your suggestion. In response to your feedback, we have added operational definitions for ambiguous variables such as immunization status, community-level poverty, and education. This addition will enhance the clarity and rigor of our study methodology.
**Reviewer’s comment:** 11. Line 109: the paragraph suggest validation of tool and it has nothing to do with quality control

**Authors’ response:** Thank you for pointing out the discrepancy. We have revised the paragraph to include relevant information regarding quality control measures. This addition ensures that the methodology section accurately reflects both the validation of the tool and the implemented quality control procedures.
**Reviewer’s comment:** 12. Line 114-115: is it 13 or 11?

**Authors’ response**: Thank you for your recommendation. During the data collection/survey year, Ethiopia had a total of 11 regions, including the two administrative cities. However, the current count has been updated to 13 regions. We were initially uncertain about which number of regions to incorporate into this study. Following your suggestion, we have decided to maintain consistency with the original count of 11 regions. This decision was made because the sampling procedure did not consider the existence of the new regions at that time.

**Reviewer’s comment:** 13. Line 151: the reference quoted is not required and is not relevant here

**Authors’ response:** dear reviewer your comment constructive. As recommended citing known, agreed and proven science might not necessary here. So, we removed the citation.
**Reviewer’s comment:** 14. Line 122: revise the heading to “data analysis” and then describe the analysis steps. Currently authors have mentioned definitions of different tests which seems like not even used for analysis or may be authors have missed the information in the results section

**Authors’ response:** Dear reviewer the title data management is revised accordingly. But, "svy set" is a command used in statistical software packages like Stata for setting up survey data analysis. It specifies survey design characteristics such as sampling weights, strata, and clusters and it is not independent analysis. Through the descriptive and analytical analysis in this study the svy command is utilized to consider hierarchical nature of the dataset. **Reviewer’s comment:** 15. Line 163-164: authors have used p-value of 0.2 or 0.05 as cutoff?

**Authors’ response:** Dear reviewer, we appreciate your constructive feedback, which has enhanced our understanding. We utilized a p-value of 0.2 to facilitate the selection process of variables from bivariable logistic regression to multivariable regression. However, we acknowledge that the sentence discussing this was not informative, ambiguous, and deemed unnecessary. Consequently, we have removed the sentences to improve the clarity and focus of our manuscript.
**Reviewer’s comment:** 16. The section needs significant improvement. Authors are requested to look into similar articles to revise the section. Sampling and study procedures needs to be strengthened

**Authors’ response:** dear reviewer we appreciate your query of clarification. The sentence was written to transfer message how we selected variables for multivariable regression from bivariable regression. It is to mean that variables in the bivariable regression with p-value <0.2 are transferred or utilized to build multivariable regression, But we agreed that this sentences is unnecessary and ambiguous by itself and we removed from the manuscript.
Results:
**Reviewer’s comment:** 17. It would be good if authors add demographic characteristics of study population first before sharing the retention rate

**Authors’ response:** Thank you for your suggestion. We agree that providing demographic characteristics of the study population before discussing the retention rate would enhance the clarity and context of the findings. We rescripted the manuscript accordingly to include demographic characteristics prior to presenting the retention rate.
**Reviewer’s comment:** 18. No p-values are given in the results section throughout

**Authors’ response:** We appreciate the reviewer's feedback. In the results section, we opted to present confidence intervals for Adjusted Odds Ratios (AOR) instead of including p-values. This decision was made due to constraints related to table cell space. We believe that providing confidence intervals offers valuable information about the precision of our estimates and allows readers to assess the significance of the results. However, following your comment, we have now included the categories of p-values for significant variables using asterisks at the footnote of the table. We believe this addition will enhance the interpretation of our findings. Thank you for understanding.
**Reviewer’s comment:** 19. Line 185-204: seems like methods section then results

**Authors’ response:** We acknowledge the reviewer's observation regarding the content of lines 185-204. We apologize for any confusion caused by the placement of methodological details within the results section. In the revised manuscript, we included that the methods section is appropriately delineated from the results, providing clarity and coherence in presenting our study's methodology and findings. Thank you for highlighting this issue.
**Reviewer’s comment:** 20. Factors associated with poor retention of vaccination cards are not mentioned

**Authors’ response:** Thank you for your comment. We appreciate your suggestion. We added Factors associated with poor retention of vaccination cards to the first paragraph of this section.

**Reviewer’s comment**: 21. Authors are requested to improve the section conveying the results clearly and adding value to scientific literature

**Authors’ response**: We acknowledge the reviewer's comment and we enhanced the clarity and value of the results section in our manuscript. We confirmed that the results are presented in a clear and concise manner, providing valuable insights that contribute to the scientific literature on the topic. Additionally, we will consider any specific suggestions or recommendations provided by the reviewer to improve this section further.
**Reviewer’s comment:** 22. Tables are missing in the manuscript, so it is difficult to see the findings or suggest corrections in table

**Authors’ response:** We appreciate the reviewer's observation. We apologize for the absence of tables in the manuscript. We warranted that the tables are included in the revised version to provide a comprehensive overview of our findings and facilitate a better understanding of the content. Thank you for bringing this to our attention.
Discussion:
**Reviewer’s comment:** 23. Line 215-216: in abstracts authors claim to have retention of VC rate across Ethiopia, now using the term sample. Is the data extracted from DHS represents entire Ethiopia? Need to improve methods section for clarity

**Authors’ response:** The data used in our study were extracted from the DHS, which is designed to be nationally representative. However, it's important to clarify that our analysis was based on a sample of the population included in the DHS dataset. We will enhance the clarity of the methods section to better explain the sampling process and the representativeness of the data used in our study. We appreciate your feedback and will ensure that the methods section accurately reflects the scope of our analysis.
**Reviewer’s comment:** 24. Line 216-218: better to mention retention rate than only mentioning the countries

**Authors’ response:** Thank you for your suggestion. We agree that specifying the retention rate rather than solely mentioning the countries would provide a clearer understanding of the context. We revised the text accordingly to include information about the retention rate in the specified countries. Your feedback is valuable, and we appreciate the opportunity to improve the clarity of our manuscript.
**Reviewer’s comment:** 25. Line 218-219: as per your study or in countries mentioned. Please rephrase

**Authors’ response:** Thank you for your comment. We rephrased lines 218-219 to ensure clarity regarding the source of the information. We appreciate your feedback and will make the necessary revisions to accurately convey the context of our study.
**Reviewer’s comment:** 26. Line 220-225: the first line says high retention in rural while rest of para contradicts the first statement. Also compared to whom retention is higher in rural? Urban or rural slum? What is considered as rural slum? Does DHS data providing information as per rural slum? How many children belong to rural slum?

**Authors’ response:** Thank you for your comments and inquiries. We apologize for any confusion caused by the inconsistency in the statement regarding retention rates in rural areas. Upon revisiting our analysis, we realized that the initial statement may not accurately reflect the findings. Our analysis indeed revealed lower retention rates in rural areas compared to urban areas. Regarding the comparison between urban and rural slum areas, we will clarify this point in our manuscript.
**Reviewer’s comment:** 27. Line 233-235: what does the current study findings suggest? Do authors found educational status as one of the factors for poor card retention? Mentioning “may be attributable” is not appropriate. Authors should provide information based on the data analysis

**Authors’ response:** Thank you for your comment and request for clarification. Following your comment, we noticed that the variable place of residency is already discussed in the paragraph above this statement and it is redundant idea here. So, we deleted this whole paragraph and agreed to continued with first paragraph bout place of residency.

**Reviewer’s comment:** 28. Line 245-247: authors should add relevant reference from literature to support the statement

**Authors’ response:** Thank you for your comment. Following your inquiry, we tried to review literatures about this personal justification or inference based on your own analysis and understanding, we are clarifying this sentence is not supported by specific literature. However, we understand that it's always beneficial to support statements with relevant research whenever possible to strengthen the credibility and validity of your argument.

**Reviewer’s comment:** 29. Line 248-250: how authors are defining poverty levels? What are the cutoffs for low and high poverty? Do authors compare the findings among different socioeconomic groups including children belonging to high socioeconomic status?

**Authors’ response:** Thank you for your fruitful comment. We have taken note of your suggestion and have included the cutoff point for community-level poverty under the operational definition section of the methodology in the manuscript. We did indeed compare immunization card retention rates among different socioeconomic groups, including children from high socioeconomic status backgrounds, and we will ensure that this comparison is clearly presented in the revised manuscript.

**Reviewer’s comment:** 30. Line 251-252: the study is not explaining crucial issues in child health. It is only about immunization card retention

**Reviewer’s comment:** We acknowledge the reviewer's observation. While our study primarily examines immunization card retention, we understand the importance of considering broader issues in child health. In the revised manuscript, we will endeavor to provide additional context on the implications of immunization card retention for overall child health outcomes and discuss potential avenues for future research to explore these aspects more comprehensively. Thank you for bringing this to our attention.
**Reviewer’s comment:** 31. Line 253-258: need to reconsider limitation of study as mentioned limitations cannot be considered as true study limitations

**Authors’ response:** We appreciate the reviewer's input regarding the perceived limitations of the study. Upon reflection, we acknowledge that while these factors are important considerations, they may not qualify as true limitations in the traditional sense. In the revised manuscript, we will provide a more nuanced discussion of these aspects, clarifying their implications for the study findings without overstating them as inherent limitations. Thank you for guiding us in refining our manuscript.
**Reviewer’s comment:** 32. Line 260: low retention compared to what?

**Authors’ response:** Thank you for pointing out this lack of clarity. In the revised manuscript, we provided context to clarify what "low retention" is being compared to, ensuring a clearer understanding for readers.
