# Supplementary material for: Retention rate of vaccination card and its associated factors among vaccinated children aged 12─23 months in Ethiopia: Multilevel logistic regression analysis
Source: PLoS One. 2024 Jul 11;19(7):e0306421. doi: 10.1371/journal.pone.0306421 (PMC11238995; doi:10.1371/journal.pone.0306421)
Supplement: Attachment — Submitted filename: Response to Reviewers.docx [file pone.0306421.s002.docx]

**Authors’ response to reviews**

# Title: Retention rate of vaccination cards and associated factors among vaccinated children aged 12-23 months in Ethiopia, multilevel logistic regression analysis

Authors:

Abiyu Abadi Tareke ([abiyu20010@gmail.com](mailto:abiyu20010@gmail.com))

Atikaw Tewabe Ayelign

Thomas Kidanemariam Yewodiaw

Enyew Woretaw Shiferaw

Habitu Birhan Eshetu

Ermias Bekele Enyew

Version: 2

Date: March 31, 2024

Point by point response for editors/reviewers’ comments

Manuscript number: PONE-D-23-21113

Dear editor/reviewer:

Dear all,

We express our profound appreciation for the insightful and productive feedback that you have provided. Your invaluable comments have significantly enriched the quality of the manuscript, and have greatly augmented our expertise in the realm of scientific paper writing. The authors have diligently considered each of the comments and queries raised by the editors and reviewers, and have responded to them in a targeted manner. Our comprehensive point-by-point rejoinders to all the comments and questions can be found in the subsequent pages. In addition, an accompanying supplementary document has been enclosed, which showcases the modifications made in detail, using the track changes feature. We also made some change to fix grammatical error in some paragraphs.

Reviewer #1:

**Reviewer’s comment:** The authors have well addressed my previous comments. I have few minor comments:

**Author’s response:** Thank you for your additional feedback on our manuscript. We appreciate you taking the time to review our work and provide these minor comments. Regarding your statement that "The authors have well addressed my previous comments", we are pleased to hear that our revisions have satisfactorily addressed your earlier feedback. It is our goal to thoroughly address all reviewer comments to ensure our work is of the highest quality.

Multilevel regression model:
**Reviewer’s comment**: 1. There are duplications of ideas, for example, the first sentence of the first (line number 183) and the second (line number 183) paragraphs, line number 213-215.

**Author’s response:** Dear Reviewer, thank you for your feedback on our manuscript. We have carefully reviewed the areas you have identified as having duplicated ideas and have made the necessary revisions to address them. Specifically, we have removed the redundant sentences from the first and second paragraphs, as well as the repetitive explanation of the multilevel analysis and its benefits for nested data structures. The revised text now reads more concisely and avoids unnecessary duplication. Additionally, we have removed the redundant information regarding the different models used in the two-tiered binary logistic regression analysis and the explanation of the fitness parameters used to evaluate the models. The revised manuscript now presents the key information in a more streamlined and cohesive manner, without compromising the clarity and comprehensiveness of the methodological approach. We believe these changes have addressed the duplication of ideas you have pointed out. Please let us know if you have any other feedback or suggestions. 
**Reviewer’s comment**: 2. Flow of ideas needs to be revised: line number 197-199 deal with null model, line number 199-203 is about model fitness, line number 203-206 states about ICC which should have been completed/placed just after the null model description before you note about the model fitness. The same is true for notes at line number 213-215.

**Author’s response:** Thank you for the additional feedback on the flow of ideas in our manuscript. We appreciate you taking the time to provide this constructive criticism, as it will help us improve the organization and coherence of the presentation. As per your suggestion, we have revised the flow of ideas to better align the discussion of the null model, model fitness, and the assessment of cluster variability using the ICC, MOR, and PCV. Specifically, we have moved the explanation of the ICC, MOR, and PCV to immediately follow the description of the null model, as this information is more logically connected. This way, the reader can better understand the rationale and methods used to assess the variability of the cluster before the discussion of the model fitness parameters. Additionally, we have streamlined the information about the different models and the criteria used to evaluate them, ensuring a more coherent and logical progression of ideas. The revised manuscript now presents the methodological approach in a more organized and reader-friendly manner, with the key concepts and analyses grouped together in a more intuitive flow.
**Discussion:
Reviewer’s comment**: 3. Line number 277, the term "In our sample" is not appropriate instead use the term: "In this survey"

**Author’s response:** Thank you for the feedback regarding the phrasing used in our manuscript. We have made the suggested change to the sentence on line 277.

The revised sentence now reads: "In this study, the majority of children (63.3%) were from rural areas, while the remaining 36.7% were from urban areas." We agree that the term "In our sample" is not as appropriate as "In this study" in this context, as it helps to clearly situate the information within the dataset being analyzed.
**Reviewer’s comment**: 4. Line number 278-281, since it is a discussion section, please search and include possible justification/reason for the observed variation!

**Author’s response:** Thank you for the feedback regarding the discussion of the observed variations in our manuscript. You make an excellent point that the discussion section should provide possible justifications or reasons for the patterns observed in the results. In response to your suggestion, we have expanded the discussion on lines 278-281 to include potential explanations for the variations in vaccination coverage between those studies, as well as across different socioeconomic groups.

**Reviewer’s comment**: 5. How do you relate the opposite direction of association of two related variables: 'being rural resident' (line number 293) and 'low community level poverty' (line number 308-309)?

**Author’s response:** Thank you for raising this important point about the seemingly contradictory associations observed in our results. You are correct in noting that the direction of the associations for "being a rural resident" and "low community-level poverty" appear to be opposite. We separated those two variables following your comment.

**Reviewer #2**

**Reviewer’s comment**: Reviewer #2: 1. p-values are still missing

**Author’s response:** Dear Reviewer, thank you for your feedback regarding the missing p-values in our manuscript. We appreciate you taking the time to thoroughly review our work and provide this constructive comment. Regarding the inclusion of p-values in our manuscript. As we mentioned in our previous response, we opted to present confidence intervals for the Adjusted Odds Ratios (AORs) in the results section, rather than including the specific p-values. This decision was made due to constraints related to table cell space, as we believe that providing the confidence intervals offers valuable information about the precision of our estimates and allows readers to assess the significance of the results. However, following your suggestion, we have now included the categories of p-values for the significant variables using asterisks at the footnote of the relevant tables.

We are sorry if our initial response may not have fully satisfied your request. As researchers, we are committed to transparency and strive to present our findings in the clearest possible way. We value your expertise and insights, and we appreciate you taking the time to provide this constructive feedback.

**Reviewer’s comment**: 2. Table 3 seems incomplete. model 2 and null model numbers are missing

**Author’s response:** As you noted, the null model, which does not include any variables, is typically not reported in the table. This is a standard practice, as the null model serves as a baseline for comparison and does not provide any substantive information beyond the intraclass correlation coefficient. Similarly, for Model 3 in Table 3, this model focuses solely on the community-level factors and does not include the individual-level variables. This design decision was intentional, as we wanted to isolate the effects of the contextual factors on the outcome of interest.

We believe that the table is complete and comprehensive in presenting the key results from the multilevel modeling approach. Dear reviewer if you have time you can highlight articles published using similar tables (<https://journals.plos.org/plosone/article?id=10.1371/journal.pone.0257664>), (<https://academic.oup.com/inthealth/article/15/5/573/7143244>)....
**Reviewer’s comment**: 3. There is a lot of room to improve language. spell check and capital letters in between the text

**Author’s response:** Thank you for your feedback. We appreciate you taking the time to review my submission and providing constructive comments to help improve the quality of my work. Regarding your suggestion to improve the language, we carefully reviewed the text and address any spelling or grammatical issues. We ensured that I am consistent with capitalization throughout the document.

**Reviewer’s comment**: 4. Authors have interchangeably used the term multilevel and multivariable regression. Suggest keeping consistency while using the term

**Author’s response:** Thank you for the feedback. You're right, we should maintain consistency in the terminology used throughout the manuscript. The terms "multilevel regression" and "multivariable regression" are often used interchangeably, but they do refer to slightly different statistical techniques.

Multilevel regression, also known as hierarchical linear modeling, is a method that accounts for the nested structure of data, such as patients within clinics or students within schools. This type of analysis allows for the estimation of both individual-level and group-level effects.

Multivariable regression, on the other hand, refers to a regression model that includes multiple independent variables to predict a single dependent variable. This is a more general term that does not necessarily imply a hierarchical data structure.
**Reviewer’s comment**: 5. add some narratives for demographic information

**Author’s response:** Thank you for the feedback. You make a good point that we should add some narrative context around the demographic information presented in the manuscript. The demographic characteristics of the study sample are an important part of understanding the generalizability and potential limitations of the findings. Providing some additional narrative description will help the reader better interpret the results. In the revised manuscript, we will include a paragraph in the Methods section that provides more details about the study population.
**Reviewer’s comment**: 6. tables should be referenced in the text

**Author’s response: T**hank you for the feedback regarding referencing the tables in the text. You're right that we should ensure the tables are properly referenced throughout the manuscript.

In the revised version, we gone through and add in-text references to each of the tables wherever the relevant information is being presented. This will help guide the reader to the appropriate table and demonstrate how the tabular data supports the narrative.
